# Genome Sequence of Streptomyces cavourensis BUU135, Isolated from Soil from a Tropical Fruit Farm in Thailand

**DOI:** 10.1128/MRA.01428-20

**Published:** 2021-05-13

**Authors:** Marut Tangwattanachuleeporn, Pattarawan Ruangsuj, Wariya Yamprayoonswat, Satapanawat Sittihan, Watthanachai Jumpathong, Montri Yasawong

**Affiliations:** a Faculty of Allied Health Sciences, Burapha University, Chonburi, Thailand; b Sensor Innovation Research Unit (SIRU), Burapha University, Chonburi, Thailand; c Program on Environmental Toxicology, Chulabhorn Graduate Institute, Chulabhorn Royal Academy, Bangkok, Thailand; d Spectroscopic and Sensing Devices Research Group, National Electronics and Computer Technology Center, National Science and Technology Development Agency, Pathum Thani, Thailand; e Program on Chemical Biology, Chulabhorn Graduate Institute, Chulabhorn Royal Academy, Bangkok, Thailand; f Center of Excellence on Environmental Health and Toxicology (EHT), Office of Higher Education, Bangkok, Thailand; University of Arizona

## Abstract

Streptomyces cavourensis BUU135 is a bacterial species isolated from the soil of a tropical fruit farm. The genome of *S. cavourensis* BUU135 comprises a gene encoding nebramycin 5′ synthase, which produces nebramycin 5′ by catalyzing the *O*-carbamoylation reaction of tobramycin. The newly sequenced 7.66-Mb draft genome of *S. cavourensis* BUU135 may contribute to the discovery of novel natural products derived from this organism.

## ANNOUNCEMENT

*Streptomyces cavourensis* is a Gram-positive bacterium found abundantly in soil (1). The type strain of *S. cavourensis* was first reported as a contaminant in marine fungal culture (2). Colonies of *S. cavourensis* are yellow or red and can produce spore chains (2). The temperature for the optimal growth of *S. cavourens*is is 28°C (2).

The bacterial strain BUU135 was isolated from soil from a tropical fruit farm in Chanthaburi Province, Thailand (12°45′31.8″N, 102°01′58.7″E). One gram of the soil sample was diluted in 10 ml of sterile phosphate-buffered saline (PBS) buffer (pH 7.4), and 100 μl of the dilution was spread onto International *Streptomyces* Project 2 medium (ISP-2). The culture was incubated at 25°C for 48 h. A single colony was picked and cultured in ISP-2 broth with shaking at 200 rpm at 25°C overnight. Genomic DNA (gDNA) from strain BUU135 was extracted by the phenol-chloroform method described by Sambrook and Russell (3). The NanoDrop spectrophotometer (Thermo Fisher Scientific, USA) was used to determine the quality and quantity of the gDNA. A sequencing library was prepared using the Ion PI Hi-Q OT2 template and Ion Plus fragment library kits. The library was placed on an Ion PI chip, and sequencing was performed using an Ion Proton sequencer (Thermo Fisher Scientific). The average read length was 83 bp. There were 6,289,427 raw reads (depth of coverage, 106×) generated by the sequencing run. The draft genome sequence of strain BUU135 was identified using the Type Strain Genome Server (TYGS) (4). The bacterial strain BUU135 was affiliated with Streptomyces cavourensis (DSM 41795) with a digital DNA-DNA hybridization (dDDH) value of 95.4% (4).

The quality of the raw reads was determined using AfterQC version 0.9.6 with default parameters (5). *De novo* genome assembly was performed using the raw reads and SPAdes version 3.13.1 in -careful mode (6). The genome assembly metric was determined using QUAST version 5.0.2 with default parameters (7). The draft genome sequence of *S. cavourensis* BUU135 comprises 7,657,683 bp in 2,754 contigs with an *N*_50_ value of 4,468 bp and 71.73% GC content. Genome quality assessment was performed using CheckM version 1.1.3 with default parameters (8). The CheckM analysis revealed that the completeness of the *S. cavourensis* BUU135 genome was 96.28%, with 5.34% contamination. Genome annotation was performed using the NCBI Prokaryotic Genome Annotation Pipeline (PGAP) with default parameters (9). The annotated genome sequence of *S. cavourensis* BUU135 contains 8,398 total genes, 8,326 protein-coding sequences, 53 tRNA genes, 16 rRNA genes, 3 noncoding RNAs (ncRNAs), and 4 CRISPR regions.

One annotated locus of the *tobZ* gene in the *S. cavourensis* BUU135 genome was determined using Prokka version 1.13.7 with default parameters (10). The *tobZ* gene encodes nebramycin 5′ synthase, an enzyme responsible for the production of nebramycin 5′ (6″-*O*-carbamoyltobramycin) by exhibiting ATP-dependent carbamoylation of tobramycin (11, 12). Nebramycin 5′ is an aminoglycoside antibiotic with activities against various types of bacterial infections (11, 12). Therefore, *S. cavourensis* BUU135 may be responsible for producing other derivatives of nebramycin 5′ with potential antimicrobial activities.

### Data availability.

The whole-genome shotgun sequence of *S. cavourensis* BUU135 has been deposited at DDBJ/ENA/GenBank under accession number JADZLU000000000 and SRA accession number SRR13123741.
